# Relevance of experimental paradigms of anesthesia induced neurotoxicity in the mouse

**DOI:** 10.1371/journal.pone.0213543

**Published:** 2019-03-21

**Authors:** Simon C. Johnson, Amanda Pan, Grace X. Sun, Arielle Freed, Julia C. Stokes, Rebecca Bornstein, Michael Witkowski, Li Li, Jeremy M. Ford, Christopher R. A. Howard, Margaret M. Sedensky, Philip G. Morgan

**Affiliations:** 1 Center for Integrative Brain Research, Seattle Children’s Research Institute, Seattle, WA, United States of America; 2 Department of Neurology, University of Washington, Seattle, WA, United States of America; 3 University of Washington School of Dentistry, Seattle, WA, United States of America; 4 Department of Pathology, University of Washington, Seattle, WA, United States of America; 5 Seattle Children's Imagination Lab, Seattle Children’s Research Institute, Seattle, WA, United States of America; 6 Department of Anesthesiology and Pain Medicine, University of Washington, Seattle, WA, United States of America; Massachusetts General Hospital, UNITED STATES

## Abstract

Routine general anesthesia is considered to be safe in healthy individuals. However, pre-clinical studies in mice, rats, and monkeys have repeatedly demonstrated that exposure to anesthetic agents during early post-natal periods can lead to acute neurotoxicity. More concerning, later-life defects in cognition, assessed by behavioral assays for learning and memory, have been reported. Although the potential for anesthetics to damage the neonatal brain is well-documented, the clinical significance of the pre-clinical models in which damage is induced remains quite unclear. Here, we systematically evaluate critical physiological parameters in post-natal day 7 neonatal mice exposed to 1.5% isoflurane for 2–4 hours, the most common anesthesia induced neurotoxicity paradigm in this animal model. We find that 2 or more hours of anesthesia exposure results in dramatic respiratory and metabolic changes that may limit interpretation of this paradigm to the clinical situation. Our data indicate that neonatal mouse models of AIN are not necessarily appropriate representations of human exposures.

## Introduction

General anesthesia has been a key feature of modern medicine for over 150 years. Although widely used and generally safe in healthy individuals, neurotoxicity related to anesthetic exposure has been reported in multiple animal models and retrospective clinical studies (reviewed in [1–4]). These studies have linked anesthetic exposure during early post-natal periods to acute anesthesia induced neurotoxicity (AIN) and later-life defects in performance in behavioral assays for learning and memory. This data has generated substantial concern regarding the risks associated with anesthesia in human neonatal and pediatric patients [1,5–7].

In humans, studies of the impact of pediatric anesthetic exposure on cognition and behavior have generally been difficult to interpret. Some retrospective studies have reported mild cognitive defects, such as impaired language skills or abstract reasoning, associated with anesthetic exposure during early childhood, while others have shown no such association [8–10]. Various confounding factors have limited the interpretation of these studies, such as differences in compared populations and an inability to separate the impact of anesthesia from the underlying pathology requiring exposure, or the stress of surgery. The General Anesthesia versus Spinal Anesthesia (GAS) and Pediatric Anesthesia & Neuro Development Assessment (PANDA) studies were designed to directly assess the neurodevelopmental impact of anesthesia while eliminating the majority of confounding effects. Reports from both of these prospective trials revealed no evidence of cognitive defects arising from short anesthetic exposures in neonates [6–11]. Similarly, the SmartTots program has recently reported that available evidence suggests brief clinical exposures do not appear to cause overt cognitive defects in humans, while also calling for increased efforts to improve reproducibility in animal studies [7]. These well-controlled, multicenter studies in children provide the best evidence to date that short clinical anesthetic exposures have minimal or no lasting impact on human cognitive development. The clinical literature is reviewed in depth elsewhere (see [1,5,12,13]). The recent review by Davidson and Sun conclude that the clinical concerns, including the FDA warning, continue to be driven primarily by pre-clinical findings [5].

The lack of measurable effects on cognition in recent multicenter human trials is reassuring, but raises important questions regarding the pre-clinical AIN literature. In particular, while the potential for exposure to anesthetic agents to damage the rodent neonatal brain is clear, the significance of pre-clinical models used to induce damage has not been carefully evaluated. With the initial aim of examining intracellular signaling changes in the AIN paradigm, we performed detailed baseline experiments to define the physiological impact of anesthesia in post-natal day 7 (P7) neonatal mice in the experimental conditions most commonly used for mouse AIN studies. Although there has been some effort to examine physiological parameters in neonatal rodents under anesthesia (see Discussion) an extensive assessment of multiple parameters has been lacking. Here, we report striking physiological and metabolic aberrations caused by common anesthesia paradigms in neonatal mice and discuss the implications of these findings as they apply to the pre-clinical AIN literature. These pathologic responses also give new insights into the potential impact of volatile anesthetics in neonates, which may lead to studies in pediatric patients that improve patient care.

## Materials and methods

### Ethics statement and animals

All experiments were approved by the Animal Care and Use Committee of Seattle Children’s Research Institute (Seattle, WA). All experiments utilize the C57Bl/6 strain, originally obtained from Jackson laboratories (Bar Harbor, ME). All treatment group assignments were randomized. Animal numbers for each dataset are noted in the associated figure legends. To increase power all baseline blood parameter datasets for P7 neonates (time = 0 for P7 neonatal anesthesia exposures) were pooled and are common between figures where baseline P7 blood data appears.

### Anesthetic exposures and respiratory rates

**Fig 1:**
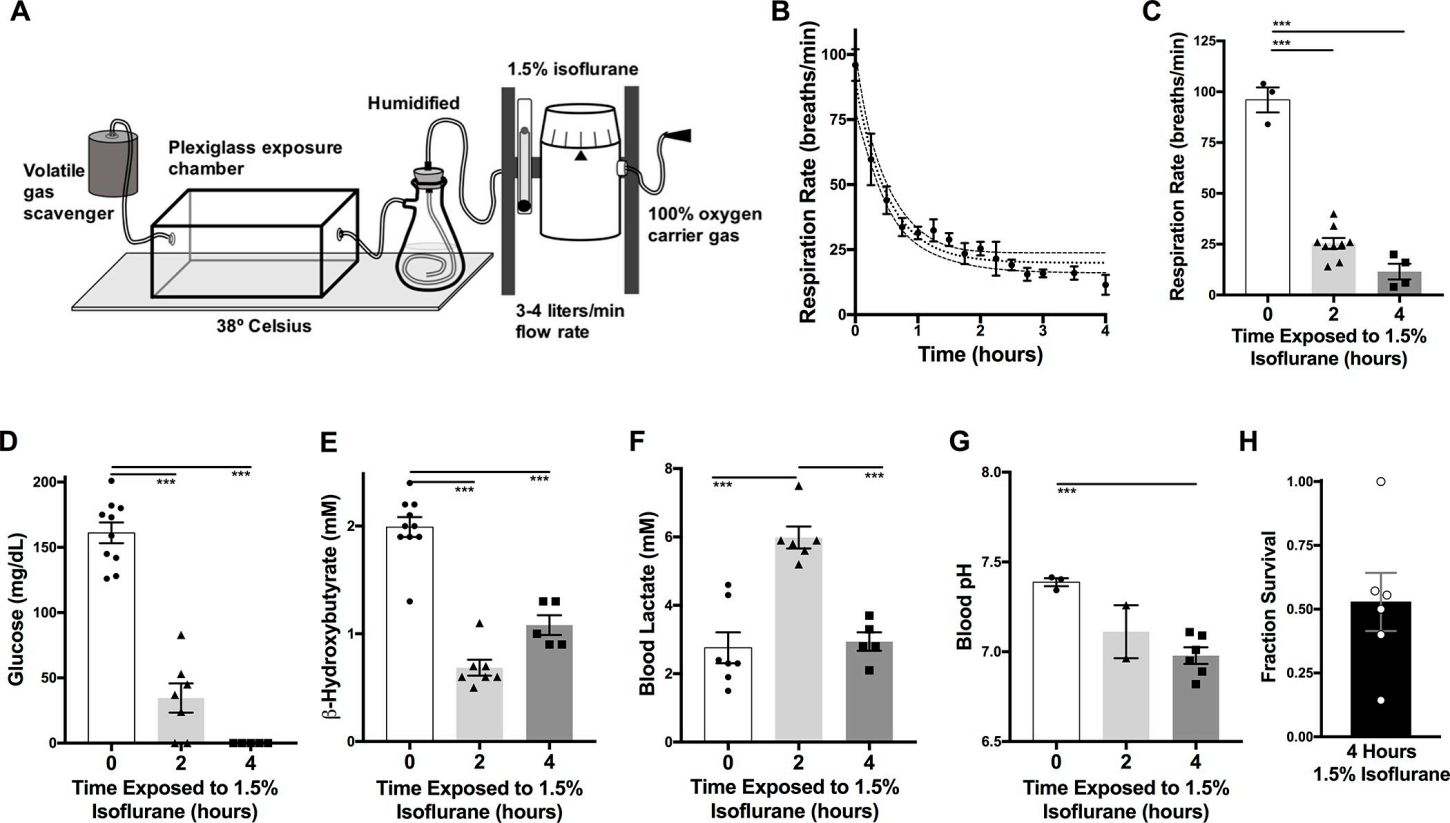
Physiological impact of prolonged anesthesia in P7 neonatal mice. (**A**) Experimental anesthesia setup. P7 neonatal mice were exposed to 1.5% isoflurane in 100% oxygen humidified air in a Plexiglass chamber, with gasses and chamber maintained at 38° C. (**B**) Respiratory rate in P7 mice as a function of time exposed to 1.5% isoflurane. Error bars depict standard error of the mean (SEM), dotted center lines depict non-linear best fit curve, dashed outer lines depict 95% confidence intervals (see Methods). n = 16 and 3 for 1.5% isoflurane and 1% isoflurane, respectively. (**C**) Comparison of respiratory rates in unexposed mice and mice anesthetized with 1.5% isoflurane for 2 or 4 hours ANOVA p-value<0.0001; ****p<0.0001 post-hoc pairwise t-test. n = 13, 7, and 5 for 0, 2, and 4 hours of exposure, respectively. (**D**) P7 mouse blood glucose concentration at baseline and at 2 or 4 hours of anesthesia with 1.5% isoflurane ANOVA p-value<0.0001; ****p<0.0001 post-hoc pairwise t-test. n = 13, 7, and 5 for 0, 2, and 4 hours, respectively. (**E**) P7 mouse blood ß-hydroxybutyrate concentration at baseline and at 2 or 4 hours of anesthesia with 1.5% isoflurane. ANOVA p-value<0.0001; ****p<0.0001 post-hoc pairwise t-test. n = 10, 7, and 5 for 0, 2, and 4 hours of exposure, respectively. (**F**) P7 mouse lactate concentration at baseline and at 2 or 4 hours of anesthesia with 1.5% isoflurane. ANOVA p-value = 0.0007; ***p = 0.0002, ****p<0.0001 post-hoc pairwise t-test. n = 7, 6, and 5 for 0, 2, and 4 hours of exposure, respectively. (**G**) P7 mouse blood pH at baseline and at 2 or 4 hours of anesthesia with 1.5% isoflurane. ANOVA p-value = 0.0007; *** = p<0.0005 post-hoc pairwise t-test. n = 3, 2, and 6 for 0, 2, and 4 hours of exposure, respectively. (**C-G**) Each data-point represents an individual animal. Error bars depict SEM. (**H**) Fraction of P7 mice surviving recovery period following exposure to 1.5% isoflurane for 4 hours. Data-points represent individual litters exposed for 4 hours, error bars represent SEM. Data represents a total of 6 litters (individual data-points) containing 4, 9, 3, 7, 7, and 5 pups, respectively. Total n = 35. *p = 0.0057 by one-sample t-test compared to a hypothetical value of zero.

We chose anesthetic conditions consistent with published mouse neonatal AIN literature (see **Results**, **Table 1**, **Fig 1A**). Isoflurane (Piramal Critical Care Inc, NDC66794-013-25) was provided at 1.5% using a routinely maintained and tested isoflurane vaporizer (Patterson Veterinary) at a flow-rate of 3–4 liters/min through a humidifier in-line. 100% oxygen was used as the carrier gas. The plexiglass exposure chamber and humidifier were pre-warmed to 38° C and maintained at this temperature throughout the exposure using a circulating water heating pad (Adroit Medical, HTP-1500), and the temperature of the heating pad was verified using a thermometer. ‘No isoflurane’ controls were treated identically to isoflurane exposed animals, including removal from parents at P7 for 4 hours and maintenance at 38° C during but exposed to room air. ‘Baseline control’ animals were not handled prior to behavioral testing aside from standard animal care. Respiratory rates were observed through direct visual observation by counting mouse breathing rate over time.

**Table 1:** Summary of neonatal mouse AIN literature.

Age	Background	Anesthetic, dose, and time	Ref.
P6-8	C57Bl/6	3% isoflurane: 2 hr/day for 3 days	[37]
P6	C57Bl/6	3% isoflurane: 6 hours	[38]
P7	C57Bl/6	Isoflurane: 0.75% for 6 hours; Lidocaine: 4 mg/kg subcutaneous; midazolam: 9 mg/kg subcutaneous	[39]
P7	(F) C57Bl/6 x (M) CD-1 F1 hybrid	1.5% isoflurane: 6 hours	[21]
P5-7	C57Bl/6	0.75% isoflurane: 4 hours 0.75% isoflurane plus midazolam: 6 hrs 1.5% isoflurane: 2 hours 2% isoflurane: 1 hour	[40]
P14	C57Bl/6	1.7% isoflurane: 35 min/day for 4 days	[41]
P6-8	C57Bl/6	3% isoflurane: 2 hours/day for 3 days	[42]
P6	C57Bl/6	2.2% sevoflurane: 2 hours per day for 3 days	[43]
P6	C57Bl/6	2% isoflurane: 2hrs/day for 3 days 8% desflurane: 2 hrs/day for 3 days	[44]
P10	129T2/SvEvMsJ x C57BL6/J F1 hybrid	3% isoflurane: 90 minutes	[18]
P6	C57Bl/6	2% sevoflurane: 6 hours	[45]
P7	C57Bl/6	1.5% isoflurane 6 hours or 150 mg/kg propofol	[29]
P6-P8	C57Bl/6	3% sevoflurane: 2 hours/day for 3 days	[46]
P7	C57Bl/6	2.9% sevoflurane: 6 hours or 150 mg/kg propofol	[47]
P7	C57Bl/6	3% sevoflurane: 6 hours (with or without 5 mg/kg thiopental or 10 mg/kg propofol)	[48]
P6-8	C57Bl/6	3% sevoflurane: 2 hours daily for 3 days	[49]
P7	C57Bl/6	1.5% isoflurane: 6 hours 2.9% sevoflurane: 6 hours 7.4% desflurane: 6 hours	[50]
P7	C57Bl/6	1.5% isoflurane: 2 hours	[51]
P6-8	C57Bl/6	3% sevoflurane: 2 hours daily for 3 days	[52]
P7	CD-1	2% isoflurane: 1 hour	[53]
P7	CD-1	2% isoflurane: 1 hour	[54]
P6-7	C57Bl/6	Sevoflurane: 3.5% for 90 min, 3% for 90 min, then 2.5% for the final 3 hours.	[55]
P7-9	C57Bl/6	1.5% isoflurane: 2 hours/day for 3 days 2.2% sevoflurane, 2 hours/day for 3 days	[56]
P7-8	CD-1 male x C57Bl/6 F1 hybrid	1.5% isoflurane: 6 hours 2.9% sevoflurane: 6 hours 7.4% desflurane: 6 hours	[57]
P7, P21, or P49	C57Bl/6	1.5% isoflurane: 6 hours	[58]
P7	C57Bl/6	0.75% isoflurane: 6 hours 1.1% sevoflurane: 6 hours	[59]

### Euthanasia and mortality during anesthesia

Animals used for tissue and blood analyses were euthanized at specific time points by decapitation as approved by our institutional IACUC. Mortality during anesthesia exposure was not expected, based on the published AIN literature, but was observed in our study. In these cases, animals stopped breathing during anesthesia, while fully anesthetized and consistent with approved isoflurane overdose euthanasia or stopped breathing at the time when anesthesia exposure ended. In the latter case, pups were decapitated to ensure humane euthanasia as per our IACUC protocol. All animals were closely monitored throughout exposures and recovery to ensure that all animals were humanely euthanized.

### Blood values

Longitudinal collection of blood data is physically impossible in P7 neonatal mice due to their extremely small size. Each blood value measurement therefore represents a single animal euthanized at the timepoint designated. Animals were rapidly euthanized by decapitation, and blood was analyzed immediately. Blood glucose was measured using a Prodigy Autocode glucose meter (product #51850–3466188). The ketone β-hydroxybutyrate (β-HB) was assessed using Precision Xtra XEGW044 meter with β-HB assay strips. Lactate was measured using Nova Biomedical assay meter (Product #40828). pH was measured using a pH Testr 10 BNC meter (Oaklon #2706055) with an Orion micro pH electrode (ThermoScientific, product #9810BN).

### Glycogen quantification

Glycogen concentration was assessed using a commercially available enzymatic colorometric assay (Sigma, MAK016) according to manufacture specifications. Briefly, flash frozen tissue samples were cryohomogenized into a frozen tissue powder on dry ice. While in a 4° walk-in cold room, a portion of the powder was rapidly moved to a microcentrifuge tube on a tared scale, the mass recorded, and the tube was placed on dry ice. This was performed carefully to prevent thawing. Deionized water was added, while on dry ice, to 20 μL water per mg tissue. The samples were boiled together for 10 minutes, with intermittent vortexing, placed quickly onto wet ice, and sonicated until homogenous with no visible pieces. At this point, an aliquot was removed for protein quantification via bicinchoninic acid (BCA) assay (ThermoFisher, #23225, as per manufacturer protocol) and later normalization against tissue protein mass. Samples were centrifuged at 13,000 rpm in a tabletop centrifuge for 10 minutes at 4°C, and the supernatant was moved into a new tube on ice.

Samples derived from liver were then further diluted by 40X to fit in the range of the standard curve, based on empirical testing. A standard curve of glycogen was prepared with the following concentrations: 1 μg/μL, 0.2 μg/μL, 0.1 μg/μL, 0.05 μg/μL, 0.01 μg/μL, 0.005 μg/μL, 0.001 μg/μL, and 0 μg/μL, each in deionized water. In PCR tubes, 4 μL of sample or standard curve were mixed 6uL Glycogen Hydrolysis Buffer. For each sample/standard, two tubes were prepared, one to serve as a “hydrolysis background control”. 1 μL of 1:5 diluted Hydrolysis enzyme mix was added to samples and standards, but not ‘hydrolysis background control samples.’ 1 μL of Hydrolysis buffer was added to “hydrolysis background controls”. Samples were incubated for 30 min at room temperature, then 8.8 μL Glycogen Development Buffer, 0.4 μL Development Enzyme, and 0.4 μL Probe were added to all tubes. Samples were then incubated for 30 min at room temperature. At the end of this incubation, samples were placed on ice and absorbance was immediately analyzed on a Nanodrop 1000 set to 460nm. Concentrations were calculated using the standard curve, with background subtracted using the ‘hydrolysis background control’ reactions, and the final concentration normalized against tissue protein mass.

### Tissue collection and processing

Following euthanasia by decapitation mouse brains were rapidly bisected down the midline. One half of each brain was flash frozen in liquid nitrogen and stored at -80 until used for protein extraction. The other half was placed in ice cold 3.8% paraformaldehyde in 1X PBS and fixed for 24 hours. Following fixation, brains were moved to a cryoprotectant solution (30% sucrose, 1% DMSO, 100 μM glycine, 1X PBS, 0.45 μm filtered, pH 7.5), and stored for over 48 hours, until the fixed tissues sank to the bottom. Tissues were then placed in OCT media (Tissue-Tek OCT compound, Sakura 0004348–01), frozen in cryoblock holders on dry ice, and stored at -80 until sectioned for staining.

### Protein extraction and Western blotting

For protein lysates, the flash frozen brain half was cryo-pulverized on dry ice until a fine powder then dissolved in 1X RIPA buffer (150 mM NaCl, 1% Triton X-100, 0.5% sodium deoxycholate, 0.1% SDS, 50 mM Tris, pH 8.0) containing protease and phosphatase inhibitor cocktail and 5mM ETDA (Thermo Scientific, 78440). This solution was briefly sonicated so fragment genomic DNA then centrifuged at maximum speed on a table-top centrifuge for 10 min at 4° C. The supernatant was moved to a new tube and protein concentration was determined using the BCA assay (Thermo Scientific, 23227).

Protein samples were run on 26 well 4–12% Tris-EDTA NuPage gels with MOPS running buffer (ThermoFisher, NP0001). 20 μg protein were loaded in each well in 1X loading dye (5X dye: 250 mM Tris- HCl, 10% SDS, 30% Glycerol, 0.05% bromophenol blue, pH 6.8) with 100 mM DTT in a total volume of 15 μL. Samples were denatured prior to loading by heating to 95 degrees for 2 min. Blots were transferred to nitrocellulose membranes (BioRad, 1704271) using a BioRad Turbo-Transfer system set to 1.3A, 25V, for 5min (BioRad optimized for low molecular weight targets).

Following transfer, blots were briefly stained with Ponceau S (Sigma, P7170) to assess quality and protein loading. Blots were blocked in 10% milk in TBS with 0.5% tween-20 for 1 hour, then primary antibody for 24–72 hours.

Initial attempts to probe entire blot area for cleaved caspase-3 all failed due to binding of antibodies to full-length caspase-3 or non-specific bands. 2 of 4 commercially available antibodies tested failed to detect a band at the correct size for cleaved caspase-3. Of the two antibodies that were successful (Cell Signaling, #9602, also used for immunostaining, and Millipore AB3623, also see text) both required cutting off the upper portion of the western blot membrane above ~25 kDa and incubating the lower portion, containing cleaved caspase-3 band, in primary antibody at a 1:100 dilution. After primary antibody incubation, blots were washed 3 times in 1X TBS, incubated for 1 hour at room temperature in goat anti-rabbit-HRP at a 1:3,000 dilution, washed 3 times, then soaked in chemiluminescent substrate (Supersignal West Pico Plus, ThermoFisher 34580) and exposed to film (CL-XPosure, ThermoScientific 34090. Actin was detected using anti-Actin- horseradish peroxidase (HRP) (Abcam, ab4990) at 1:5,000 on the upper half of the blot. Successful detection of cleaved caspase-3 required extended exposures (film exposed over a weekend). Exposed films were scanned then analyzed using Image J.

### Immunofluorescent staining for cleaved caspase-3

Frozen brains in cryoblocks were cut at 50 μm thickness using a Leica CM30505 cryostat set at -40 degrees C. Slices were moved to 1X PBS and stored at 4 degrees C until used for staining. Prior to staining, slices were mounted on slides and briefly dried to adhere.

Antibody staining was performed as follows: slides were first treated for 30 min in a gentle antigen retrieval and permeabilization buffer (0.05% Triton X-100, 50 μM digitonin, 10 mM Tris-HCl, 1 mM EDTA, pH 9.0) using the double-boiler method with the lower chamber of water heated to a rapid boil. After 30 minutes the slide chambers were removed from the boiler and slides were allowed to cool to room temperature in the antigen retrieval/permeabilization buffer. To reduce formaldehyde induced background fluorescence, slides were treated with sodium borohydride in ice cold PBS, added at 1 mg/mL immediately before incubation, on ice for 30 min, then moved to 10mM glycine 1XPBS, pH 7.4, for 5 min at room temperature. Lipid background fluorescence was then blocked by incubating slides in 0.2 um filtered Sudan Black B solution (5 mg/mL in 70% ethanol) for 30 min at room temperature with very gentle motion on a bench-top rotary shaker. Slides were then rinsed twice, 5 min each, in 1X PBS. Excess fluid was wiped from the slide and the tissue was circled using Liquid Blocker PAP pen (Fisher Scientific, NC9827128) to hold staining solutions. Slides were blocked for 15 min at room temperature in 1X PBS with 10% rabbit serum (Gibco, 16120–099—all conjugated primary antibodies were produced in rabbit) then stained overnight at 4° C in a mixture of rabbit anti-caspase-3-Alexa647 (Cell Signaling, D3E9, #9602S) at 1:200, rabbit anti-NeuN-Alexa594 (Abcam, #ab207279) at 1:200, and DAPI (Sigma, D9542) at 1 μg/mL. The following day the slides were washed 3X 5 min in 1X PBS then mounted in aqueous mounting media with aqueous anti-fade (90% glycerol, 0.5% n-propyl gallate, 20mM Tris-HCl, pH 8.0), sealed with coverslips, and stored at 4° C protected from light until imaging.

### Microscopy

All compared images were collected on the same day in the same imaging session using the same imaging parameters.

Slices stained for cleaved caspase-3, NeuN, and DAPI were imaged on a Zeiss LSM 710 confocal microscope. Images of hippocampus were collected using a 10X dry objective at 0.6X optical zoom, resulting in images of 1417 x 1417 microns in physical area. All images of hippocampus were collected at 4096 x 4096 resolution, with 8–16 line averages and a line scan speed of 6–7. The DAPI channel was set to 10-micron optical section thickness, the NeuN-Alexa594 and cleaved caspase-3-Alexa647 were both set to 15-micron thickness. DAPI was excited at 405 nm, with emission light collected using a sliding filter with the range setting at 413–530 nm. NeuN (Alexa594) was excited with a 543 nm laser, emissions collected at 569–665 nm. caspase-3 (Alexa647) was excited at 633 nm and emitted light was collected at 654–698 nm.

### Assessment of cognitive function by the Barnes maze test

A Barnes maze was built by our in-house fabrication group, Seattle Children's Imagination Lab, using publicly available design specifications for commercially available mazes. Our Barnes maze was constructed using a 0.5-inch-thick, 36-inch diameter, high-density polyethylene (HDPE) disk purchased from TAP Plastics, a local fabrication supplier (https://www.tapplastics.com/). A trammel, protractor, and ruler were used to mark 20 equally spaced (18°) holes, each 16 inches from center of disk, and a drill press and hole saw were used to drill these holes. PVC caps cut to 0.5 inches were adhesively welded into the holes using TAP Poly-Weld Adhesive. The ‘escape’ hole also had a short segment of PVC inserted but this was open at the bottom rather than capped. Razor blades and a palm sander were used to tidy the surface until the entire platform was smooth and flush.

The assembled maze was placed on a platform raising it 4-feet from the ground in a small, well-lit behavior room with no windows and protected from noise. The maze was placed near a corner with unique placards placed at maze-top height on two walls and a bright lamp at about 6-foot height in the corner. The maze was kept in the same orientation at all times. Initially a small, directly attachable ‘exit’ box was used, but in tests we found mice were afraid of the small enclosure and would not return after the first successful trial. We ultimately replaced this with a Kaytee CritterTrail 3" Elbow Tube which led to a standard mouse housing unit (from our animal facility) which was replaced for each trial. The entire maze surface and elbow tube were thoroughly cleaned with 10% bleach solution [14] after every run and a clean empty cage unit used for every trial to prevent odor mediated cues from disrupting the assessment of learning and memory.

To assess learning and memory we utilized a standard Barnes maze paradigm with a slightly modified trial number to prevent acclimation. Specifically, we tested mice three times on day 1 of week one, twice on days 2 and 3 of week one, then twice on day 1 of week 2, followed by once each on day 1 of week 3 and 6 (see Results). In our assay optimization we found that additional tests, or additional test days, resulting in rapidly worsening maze times, which we interpreted to indicate acclimation to the testing environment and loss of motivation to exit the maze. An assessment of all mice tested using our paradigm shows that both learning (reduction in trial time from trial 0) and memory (maintenance of low trial time) behaviors were successfully observed in this assay (see Results), while only mild acclimation effects were observed and only in males on run 3 of day 1. In addition, to maximize the utility of the assay, we added a test at 6 weeks after the first run to assess long-term memory.

### Chemical reagents

Unless stated otherwise, all chemical reagents were purchased from Sigma-Aldrich.

### Loss of righting reflex

P7 neonatal mice were placed in an anesthesia chamber as in Fig 1, except that the humidifier was omitted for these brief exposures. Anesthetic flow rate was set to 4 liters per minute for rapid gas replacement in the plexiglass chamber. Animals were exposed incrementally to increasing doses of anesthetic for 10 min each dose, with loss of righting reflex assessed at the end of the 10 minutes. Animals were tested in groups of 4 or less, with a total of 13 individual animals tested with isoflurane and 15 individual animals tested with sevoflurane.

### Statistical analyses

All statistical analyses performed using GraphPad Prism version 7.0a. Nonlinear regression and 95% confidence intervals were calculated using a one-phase decay model with least squares regression, no weighting, and no special handling of outliers. All replicate y values were considered individual points, and medium convergence criteria were selected. Asymmetrical 95% confidence intervals are plotted as confidence bands.

All data collected are presented in the manuscript, no data-points were omitted.

All other datasets in Fig 1 were first compared by one-way ANOVA (p-values detailed in Fig legend) followed by post-hoc pairwise two-sided t-test comparisons.

**Fig 2:**
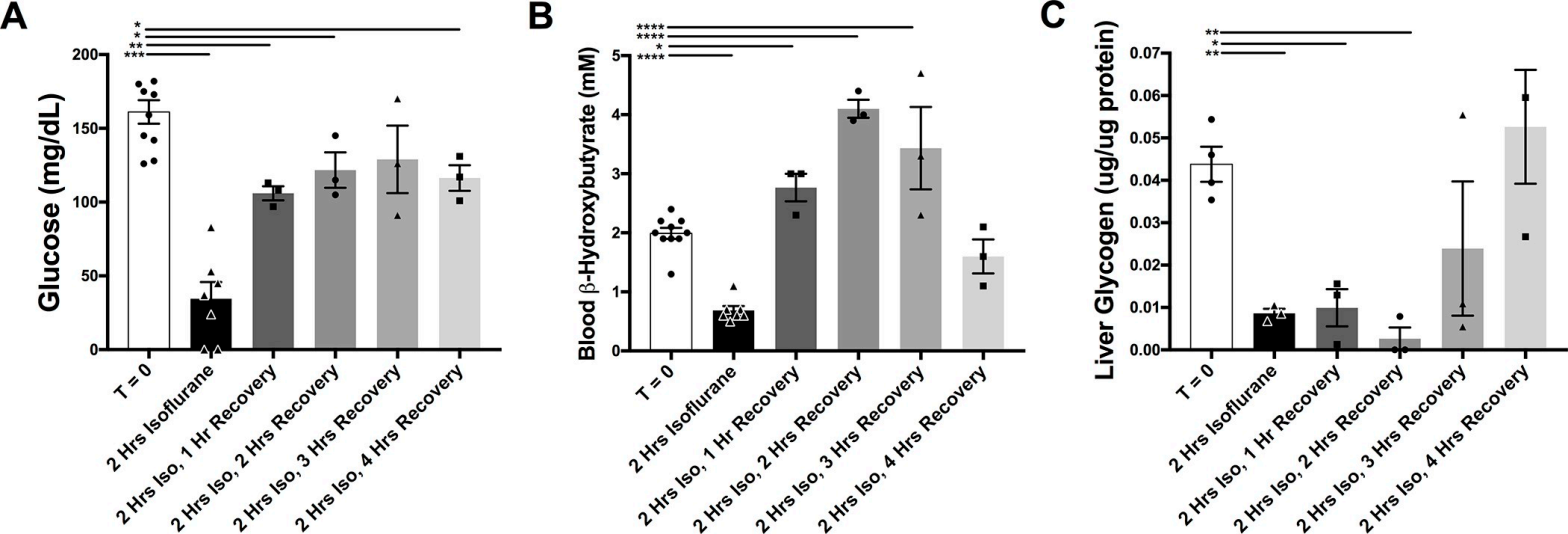
Metabolic recovery timeframe in P7 neonates anesthetized for 2 hours. (**A**) P7 mouse blood glucose concentration at baseline, at 2 hours of anesthesia with 1.5% isoflurane, and at 1 to 4 hours of recovery from 2 hours of anesthesia (baseline and 1-hour timepoints also in **Fig 1**). ANOVA p-value<0.0001; *p<0.05, **p<0.005, ***p<0.0005 by post-hoc pair-wise t-tests. n = 10, 7, 3, 3, 3, and 3 for baseline, 2 hours exposure, and 1, 2, 3, and 4 hours post-exposure, respectively. (**B**) P7 mouse blood ß-hydroxybutyrate concentration at baseline, at 2 hours of anesthesia with 1.5% isoflurane, and at 1 to 4 hours of recovery from 2 hours of anesthesia. ANOVA p-value<0.0001; *p<0.05, ****p<0.0001 in post-hoc pair-wise t-tests. n = 10, 7, 3, 3, 3, and 3 for baseline, 2 hours exposure, and 1, 2, 3, and 4 hours post-exposure, respectively. (**C**) Liver glycogen concentration at baseline, at 2 hours of anesthesia with 1.5% isoflurane, and at 1 to 4 hours of recovery from 2 hours of anesthesia. ANOVA p-value<0.0001; *p<0.05, **p<0.005, ***p<0.0005 in post-hoc pair-wise t-tests. n = 4, 3, 3, 3, 3, and 3 for baseline, 2 hours exposure, and 1, 2, 3, and 4 hours post-exposure, respectively. Each data-point represents an individual animal; error bars depict SEM. Baseline and 1-hour timepoints in (**A**) and (**B**) also in **Fig 1**.

**Fig 3:**
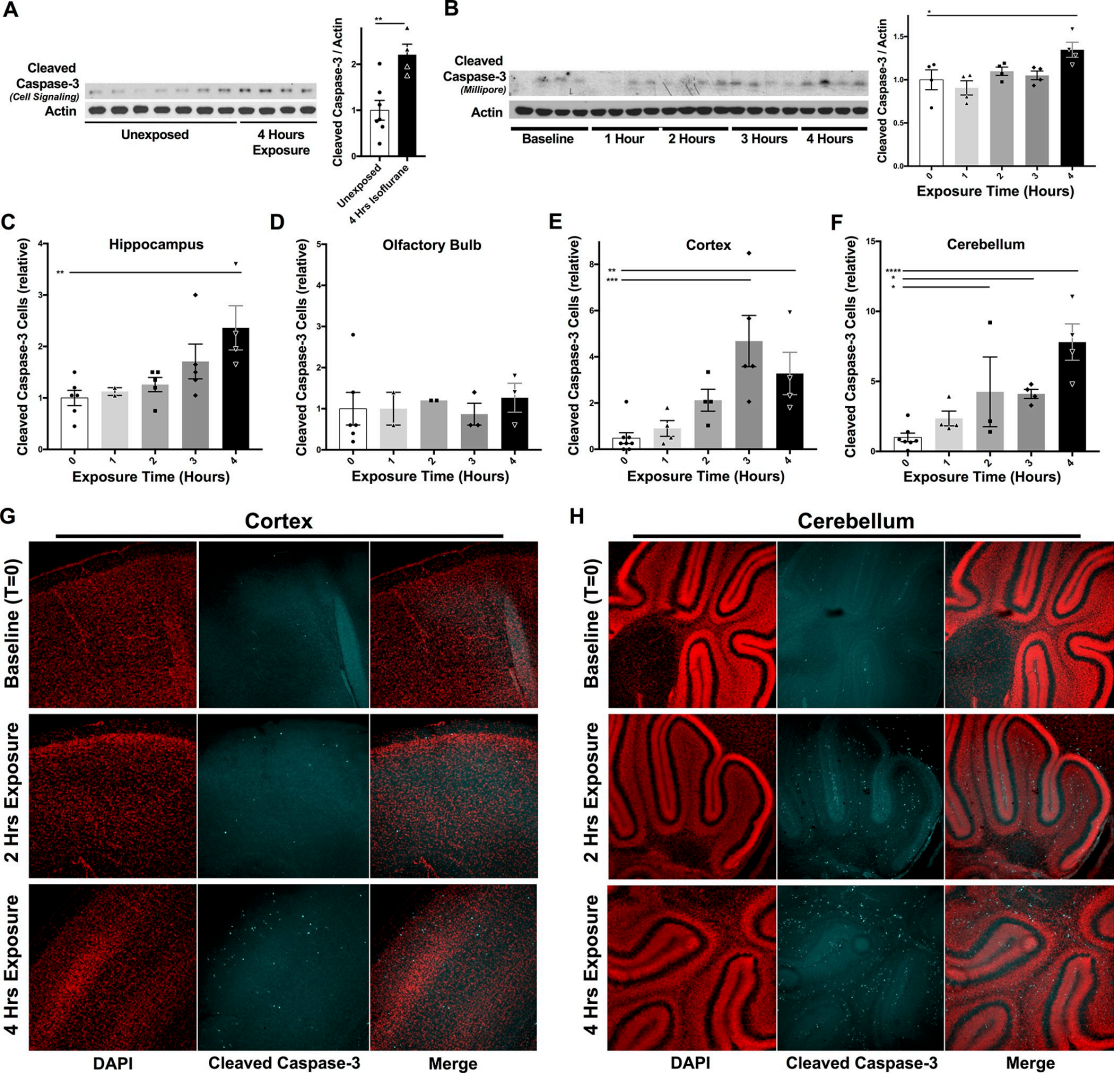
Anesthesia induced apoptosis in P7 neonatal mice. (**A**) Western blot probing for cleaved caspase-3 in brain lysates from control P7 mice (unexposed) and P7 mice exposed to 1.5% isoflurane for 4 hours. **p<0.005, pair-wise t-test. n = 7 and 4 for baseline and exposed, respectively. (**B**) Western blot probing for cleaved caspase-3 in brain lysates from P7 mice exposed to 1.5% isoflurane for 0 to 4 hours. ANOVA p-value<0.0008; **p<0.005, post-hoc pair-wise t-test. n = 4 at each timepoint. (**C-F**) Quantification of cleaved caspase-3 positive cells in the hippocampus (**C**), olfactory bulb (**D**), cortex (**E**), and cerebellum (**F**) of P7 neonatal mice exposed to 1.5% isoflurane for 0–4 hours. One-way ANOVA: hippocampus: *p<0.02; olfactory bulb: non-significant; cortex ***p = 0.0006; cerebellum *** p = 0.0007. In each panel *p<0.05, **p<0.005, ***p<0.0005 in post-hoc pair-wise t-tests. Error bars represent SEM. (**G-H**) Representative confocal images of immunofluorescent staining for cleaved caspase-3 in the cortex (**G**) or cerebellum (**H**) of animals exposed to 1.5% isoflurane for 0, 2, or 4 hours. The red channel represents DAPI, a fluorescent DNA binding molecule. The cyan channel represents the anti-cleaved Caspase-3 antibody directly conjugated to AlexaFluor647. Additional details regarding replicate numbers in cleaved caspase-3 staining in Methods.

Datasets in Figs 2 and 3 were first compared by one-way ANOVA (p-values detailed in Fig legend) followed by post-hoc pairwise two-sided t-test comparisons.

In Fig 3, a total of 8, 4, 5, 5, and 5 animals were collected for baseline, 1, 2, 3, and 3 hour 1.5% isoflurane exposure cleaved caspase-3 staining. All samples were blinded; all imaging and quantification were performed on blinded specimens. Samples with poor cut quality due to tissue folding or lack of structure of interest (for example, olfactory bulb and cerebellum, which are sometimes lost during slice handling), were not imaged. The number of replicates per region varied based on available quality stained sections, but all quality samples were imaged and analyzed. Data-points in Fig 3 represent biological replicates; n = 6, 2, 5, 5, and 4 for olfactory bulb; n = 6, 2, 2, 3, 3 for cortex; n = 8, 4, 4, 5, 4; and n = 7, 4, 3, 4, 4 for cerebellum.

**Fig 4:**
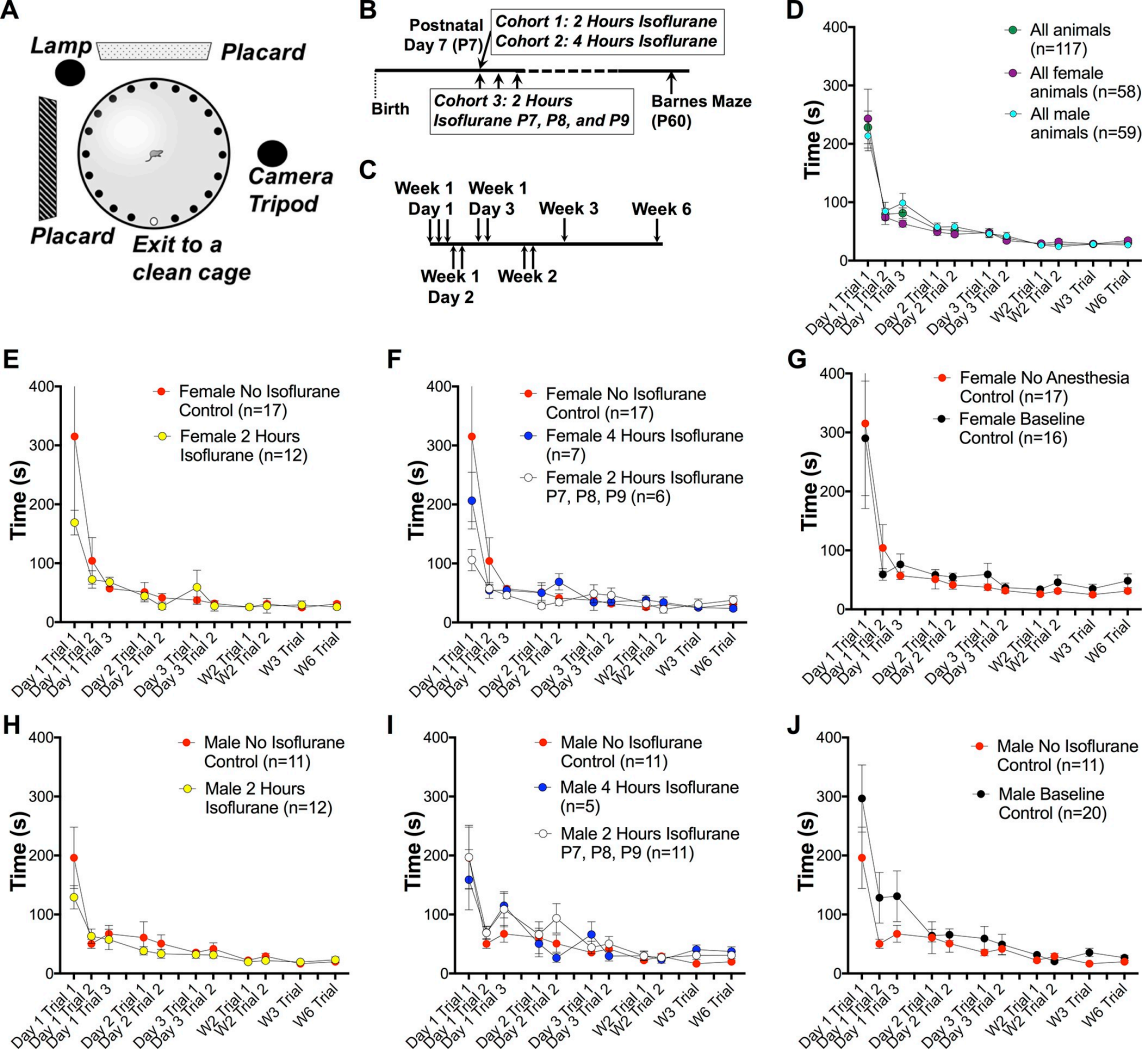
Cognitive outcomes in anesthesia exposed P7 neonatal mice. (**A**) Barnes maze setup and Barnes maze testing paradigm (see Methods for details and specifications). (**B**) Timeline of anesthesia treatment and Barnes maze assessment and anesthesia paradigms explored in this study. (**C**) Barnes maze paradigm utilized in this study. (**D**) Composite data from all mice from all treatment cohorts. (**E, H**) Assessment of female (**E**) and male (**H**) animals treated with 2 hours of anesthesia with 1.5% isoflurane mice compared to mock treated controls (see Methods). (**F, I**) Assessment of female (**F**) and male (**I**) animals treated with 4 hours of anesthesia with 1.5% isoflurane or treated 2 hours of 1.5% isoflurane daily for three days compared to mock treated controls (see Methods). (**G, J**) Assessment of female (**G**) and male (**J**) animals given mock treatment compared to animals receiving no handling (aside from routine animal maintenance) prior to behavioral assessment (see Methods). Animal numbers are listed in every panel; in all panels data points represent the mean, error bars represent SEM. Row by row comparisons by pairwise t-tests yielded no significant differences (by p-value <0.05 cutoff) at any time point in any paradigm, even when no multiple testing correction was applied and thus the least stringent criteria for statistical significance used.

Data in Fig 4 were analyzed using row by row comparisons via pairwise t-tests. We compared treatment groups to both baseline controls (untreated at P7) and ‘no isoflurane’ controls (mock treatment). No significant differences (by p-value <0.05 cutoff) at any time point in any paradigm, even when no multiple testing correction was applied, thus the least stringent criteria for statistical significance, was used.

## Results

### Anesthetic approach

We first searched the literature to understand the parameters used in studies of AIN in rodents (see **Table 1**). We found that although conditions vary widely between individual reports, the majority of studies expose C57Bl/6 mice at around P7 to 2–6 hours of anesthesia. We designed our anesthetic exposure to match the most representative published AIN studies. Accordingly, isoflurane was provided in 100% oxygen at a high flow-rate of 3–4 liters/min with a humidifier in-line, and the exposure chamber, humidified gas, and chamber were pre-warmed to, and maintained at 38° C using a circulating water heating pad (**Fig 1A**, see **Methods** for additional details). We found 1.5% isoflurane provides effective anesthesia in P7 neonatal mice, as determined by loss of response to tail clamp. 1% isoflurane failed to anesthetize P7 pups, therefore we did not include this sub-anesthetic dose in our studies. Since 1.5% isoflurane is the most commonly used concentration, we exposed P7 pups to 1.5% isoflurane for 2 or 4 hours.

### Physiological impact of extended anesthesia exposure in P7 neonatal mice

To query the clinical significance of murine neonatal AIN paradigms we assessed physiological parameters associated with anesthetic exposure.

Exposure to 1.5% isoflurane in P7 neonatal mice causes progressive respiratory depression, leading to severely reduced respiratory rate (RR) at both 2- and 4-hours (**Fig 1B and 1C**). Mean RR in P7 neonatal mice at rest is ~96 breaths/min; by 2-hours, RR has dropped to ~25.4 breaths/min, further decreasing to 11.5 breaths/min by 4-hours.

We next examined circulating metabolites to assess the impact of extended anesthesia on metabolic status. Baseline glucose in P7 mice is 161 mg/dL; by 2-hours of 1.5% isoflurane glucose has fallen by ~80% to ~35 mg/dL Glucose fell below the limits of detection (<5 mg/dL) by 4-hours of exposure (**Fig 1D**). The circulating ketone β-hydroxybutyrate (β-HB) was also depleted by 2-hours isoflurane exposure, to approximately 35% of baseline (**Fig 1E**). Of note, glucose infusion did not attenuate the depletion of β-HB (data not shown).

VAs, including isoflurane, impair mitochondrial respiration through inhibition of complex I of the mitochondrial electron transport chain [15,16]. This can increase anaerobic metabolism of glucose to lactate. Respiratory depression, as observed here (**Fig 1B and 1C**), can also increase anaerobic glucose metabolism, increasing lactate. In the setting of increased anaerobic glucose metabolism, accumulating lactate can lead to metabolic acidosis. Therefore, we measured circulating lactate at 2- and 4-hours of isoflurane exposure. Blood lactate rose by 2-hours to 6.0 mM, from a baseline of 2.8 mM (**Fig 1F**). Lactate returned to baseline by 4-hours, a reduction presumably the result of depleted substrate (glucose). In agreement with the metabolic and respiratory changes, blood pH progressively dropped; by 4-hours of treatment, average blood pH was 6.98 (**Fig 1G**). As with the metabolic and respiratory parameters, this shift in blood pH would not be tolerated in a clinical setting.

### Mortality

All animals treated for 2-hours recovered from the anesthetic exposure. In contrast, while the majority of animals (>90%) treated with 1.5% isoflurane for 4-hours were still alive (judged by visible respiration) at the 4-hour time-point, substantial mortality occurred within a few minutes of removal from isoflurane. In some animals, tremors or brief tonic seizures were observed during the immediate recovery time, presumably resulting from the substantial combined physiological stress of absent blood glucose, depleted ketones, and acidosis. In total, approximately 50% of pups treated for 4-hours and allowed to recover did not survive the recovery period: from 6 independent litters, 17 survived and 18 died (see **Fig 1H**).

### Recovery time

Many AIN reports (see references in **Table 1**) suggest that extended or repeated anesthesia may lead to lasting cognitive defects in mice (although, critically, other reports have found no cognitive effects, see [1] and **Discussion**). One particularly thorough investigation of AIN in mice reported cognitive defects only at extremely high VA concentrations, after lengthy exposures, or in animals exposed repeatedly– 3 times during the same day [17]. Given the severe metabolic and respiratory impact of anesthesia for even 2-hours, we considered the time necessary for P7 mice to metabolically recover after isoflurane exposure. Blood glucose remains low even at 4 hours after a 2-hour exposure (**Fig 2A**). Recovery of circulating ketone ß-HB takes 4 hours, with ß-HB overshooting normal baseline levels at 1, 2, and 3 hours of recovery and taking time to stabilize (**Fig 2B**). Finally, quantification of liver glycogen demonstrated a depletion of hepatic glycogen stores by 2 hours of exposure which is not replenished until 4 hours of recover (**Fig 2C**).

### Timeframe of anesthesia induced neurotoxicity

Increased CNS apoptosis has also been reported in animals anesthetized with VAs for four or more hours. This is typically shown using Western blotting or antibody-based tissue staining for cleaved caspase-3, an effector of apoptosis active only following pro-apoptotic stimuli. To assess induction of apoptosis, we first probed cleaved caspase-3 by western blotting using whole brain lysates (see **Methods** for tissue collection and processing) from animals exposed to 1.5% isoflurane for 0 or 4 hours, euthanized at the 4 hour timepoint (since the 4 hour samples were taken immediately following anesthetic exposure some of the mice studied would most likely have died during recovery, based on our survival data in **Fig 1H**). We observed an induction of apoptosis at this 4-hour time-point, as reported by others (**Fig 3A**). Worth noting, we observed that Western blots required considerable attention to detail, as 2 of the 3 commercially available antibodies only detected proteins whose molecular weights did not match that of cleaved caspase 3 (see **Methods**).

Given our observations regarding RR, metabolism, blood pH, and survival, we next determined when, in relation to these changes, induction of CNS apoptosis is detectable in P7 mice. To establish a time-course relating the systemic changes to brain apoptosis we collected a set of animals exposed to 1.5% isoflurane for 0, 1, 2, 3, or 4 hours. Using this tissue set we observed that cleaved caspase-3 levels increase as a function of exposure time but, critically, induction of apoptosis was not evident until the 4-hour time-point, where cleaved caspase-3 levels in whole brain were ~50% higher than at baseline (**Fig 3B**).

We also assessed the number of apoptotic cells by probing for cleaved caspase-3 using immunofluorescent staining and confocal imaging, focusing on the hippocampus (**Fig 3C–3F**, see **Methods**). As stated above, commercial antibodies required careful selection and extensive optimization (see **Discussion**) to rigorously detect only cleaved caspase. Apoptosis induction was statistically significant in mice anesthetized for 4 hours in hippocampus, cortex, and cerebellum. Modest induction was observed at 2 hours in cerebellum. Induction was non-significant before 2-hours in every region.

### Impact of extended or repeated anesthesia exposure on learning and memory in P7 neonatal mice

Given our metabolic findings and apoptosis data we sought to determine if a 2-hour exposure to 1.5% isoflurane leads to cognitive defects. The metabolic state at 2 hours, although grossly abnormal, was less severe than the 4-hour exposure. To test this, we used the Barnes maze paradigm for learning and memory to test young adult (P60) animals that had been exposed to isoflurane or control conditions at P7 (see **Methods** for details) (**Fig 4A–4C**). To maximize the utility of this assay we added a final trial, 3 weeks after the closest previous trial on week 3, to assess longer-term memory. We found that a 2-hour anesthesia exposure had no impact on learning, short term memory, or long-term memory in either female (**Fig 4E**) or male (**Fig 4H**) animals.

We sought to test the published long-term (4-hour) and repeated (2 hours daily for 3 days) exposure paradigms. Given our metabolic recovery findings (**Fig 2**), we chose not to consider a 3-exposure per day paradigm as it is overtly not relevant to clinical exposures. As in the 2-hour treatment, we observed no detectable cognitive effects in either female (**Fig 4F**) or male (**Fig 4I**) pups as a result of 2-hour exposures repeated for 3 consecutive days or to a single 4-hour exposure, which was lethal to 50% of animals.

The majority of mouse AIN reports provide little description of their control animals, so we considered the possibility that our ‘no isoflurane’ treatment (removal from parents for the duration of the exposure time, see **Methods**) was not representative of studies where a cognitive effect was observed; it is possible that in at least some studies the published ‘control’ animals were not handled prior to behavioral testing. To address this possibility, we included a ‘baseline control’ group, where animals did not receive a ‘mock’ exposure; these animals were not handled, aside from normal care, until behavioral testing at P60. We found no differences in female (**Fig 4G**) or male (**Fig 4I**) ‘baseline control’ mice compared to any of the anesthesia paradigms tested (see statistical analyses in **Methods**).

### Impact of equipotent sevoflurane

**Fig 5:**
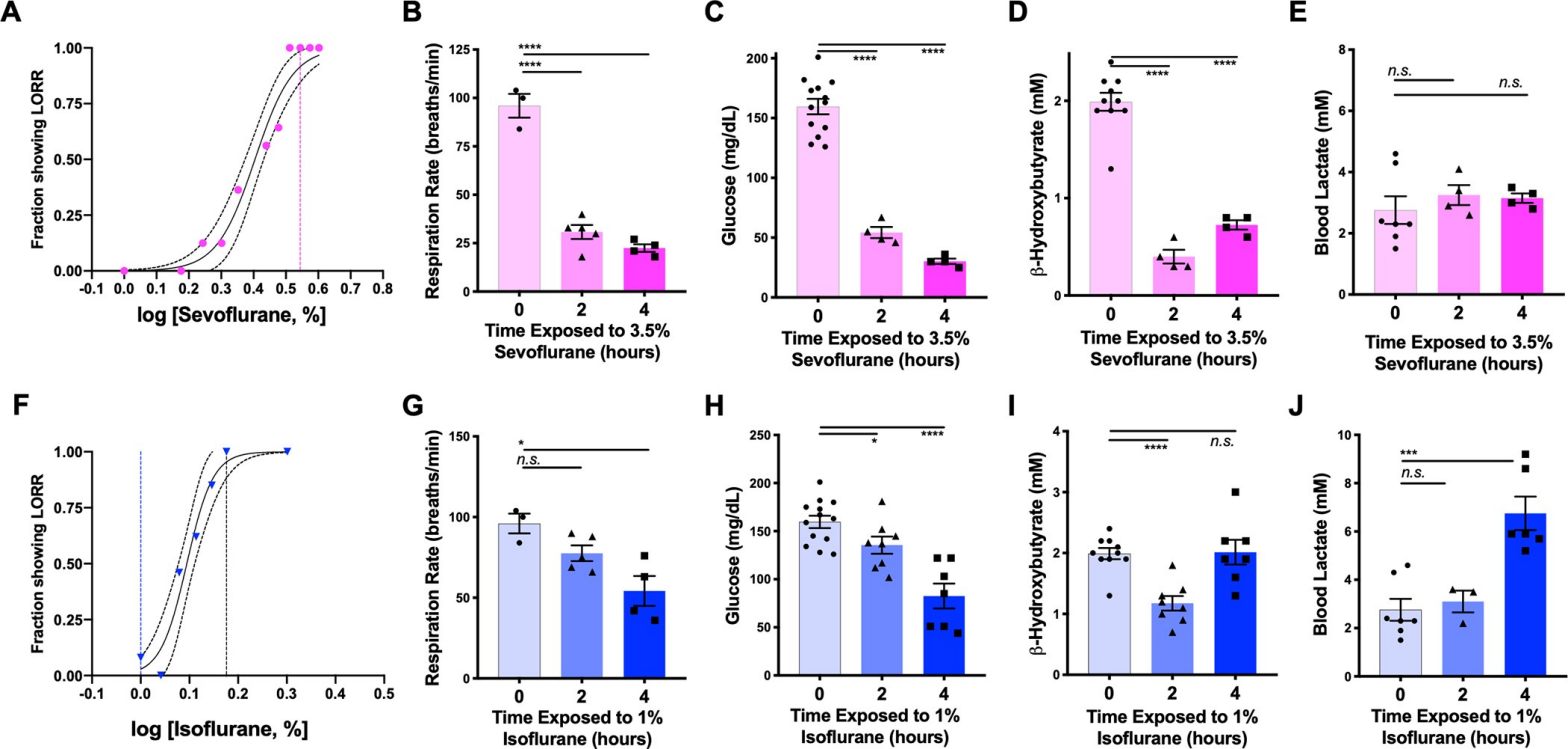
Physiological impact of equipotent sevoflurane or low dose isoflurane in neonatal mice. (**A**) Loss of righting reflex by dose of sevoflurane in P7 neonatal mice. EC50 for sevoflurane was found to be ~2.6% (95% CI = 2.44%-2.73%). Equipotent EC95 for isoflurane and sevoflurane were ~1.5% and ~3.5%, respectively (see panel F). The dashed vertical line marks 3.5%, the concentration used for data in panels B-E. (**B**) Respiratory rate of P7 neonatal mice as a function of time exposed to 3.5% sevoflurane. ANOVA p-value<0.0001; ****p<0.0001 post-hoc pairwise t-test. n = 3, 5, and 4 for 0, 2, and 4 hours, respectively. (**C**) P7 mouse blood glucose concentration at baseline and at 2 or 4 hours of anesthesia with 3.5% sevoflurane. ANOVA p-value<0.0001; ****p<0.0001 post-hoc pairwise t-test. n = 13, 4, and 4 for 0, 2, and 4 hours, respectively. (**D**) P7 neonatal mouse blood ß-hydroxybutyrate concentration at baseline and at 2 or 4 hours of anesthesia with 3.5% sevoflurane. ANOVA p-value<0.0001; ****p<0.0001 post-hoc pairwise t-test. n = 10, 4, and 4 for 0, 2, and 4 hours of exposure, respectively. (**E**) P7 mouse blood lactate concentration at baseline and at 2 or 4 hours of anesthesia with 3.5% sevoflurane. No significant differences by ANOVA or post-hoc t-test. (**F**) Loss of righting reflex by dose of isoflurane in P7 neonatal mice. EC50 for isoflurane was found to be 1.25% (95% CI = 1.21%-1.29%). The dashed vertical lines mark 1.5% and 1%, the doses used in previous figures and in panels G-J, respectively. (**G**) Respiratory rate of P7 neonatal mice as a function of time exposed to 1% isoflurane. ANOVA p-value = 0.01; *p = 0.018 post-hoc pairwise t-test. n = 3, 5, and 4 for 0, 2, and 4 hours, respectively. (**H**) P7 mouse blood glucose concentration at baseline and at 2 or 4 hours of anesthesia with 1% isoflurane. ANOVA p-value<0.0001; ****p<0.0001 post-hoc pairwise t-test. n = 13, 7, and 8 for 0, 2, and 4 hours, respectively. (**I**) P7 neonatal mouse blood ß-hydroxybutyrate concentration at baseline and at 2 or 4 hours of anesthesia with 1% isoflurane. ANOVA p-value = 0.0003; ****p<0.0001 post-hoc pairwise t-test. n = 10, 7, and 8 for 0, 2, and 4 hours of exposure, respectively. (**J**) P7 neonatal mouse blood lactate concentration at baseline and at 2 or 4 hours of anesthesia with 1% isoflurane. ANOVA p-value = 0.0004; ***p = 0.0004 post-hoc pairwise t-test. n = 7, 3, and 6 for 0, 2, and 4 hours of exposure, respectively.

While the majority of neonatal mouse AIN studies have used isoflurane to induce CNS death and behavioral defects (see Table 1), sevoflurane is a newer volatile anesthetic which is preferred in clinical pediatric practice and is becoming more common in pre-clinical research. To determine whether sevoflurane exposure induces physiological changes similar to those observed in neonates exposed to 1.5% isoflurane we repeated the extended duration exposures using sevoflurane at an equipotent dose, 3.5% (see Fig 5A and 5F). As with isoflurane, extended exposure to sevoflurane resulted in a progressive respiratory depression (Fig 5B), hypoglycemia (Fig 5C), and hypoketonemia (Fig 5D). Intriguingly, sevoflurane did not induce the increased blood lactate seen with isoflurane treatment, although we cannot rule out the possibility that there was a transient increase in lactate at some time between the measured points (Fig 5E).

### Anesthetic dose

To determine whether it is possible to avoid the metabolic effects of anesthesia by reducing the concentration of volatile gas we next tested a lower dose of isoflurane, 1%. Importantly, 1.5% isoflurane and 3.5% sevoflurane are approximately EC95 for loss of righting reflex (LORR) in P7 neonatal mice (Fig 5A and 5F). while 1% isoflurane is well below the EC50 for P7 neonatal animals. Extended 1% isoflurane exposure resulted in a slight respiratory depression by 4 hours (Fig 5G) with hypoglycemia (Fig 5H), a transient reduction in circulating ß-hydroxybutyrate (Fig 5I), and an increase in blood lactate by 4 hours of exposure (Fig 5J). While the metabolic changes at 1% isoflurane were less severe than those resulting from exposure to 1.5% isoflurane, these data indicate that extended exposure to volatile anesthetic agents, even at sub-anesthetic doses, results in significant physiological disturbances in P7 neonatal mice.

### Age range of sensitivity

**Fig 6:**
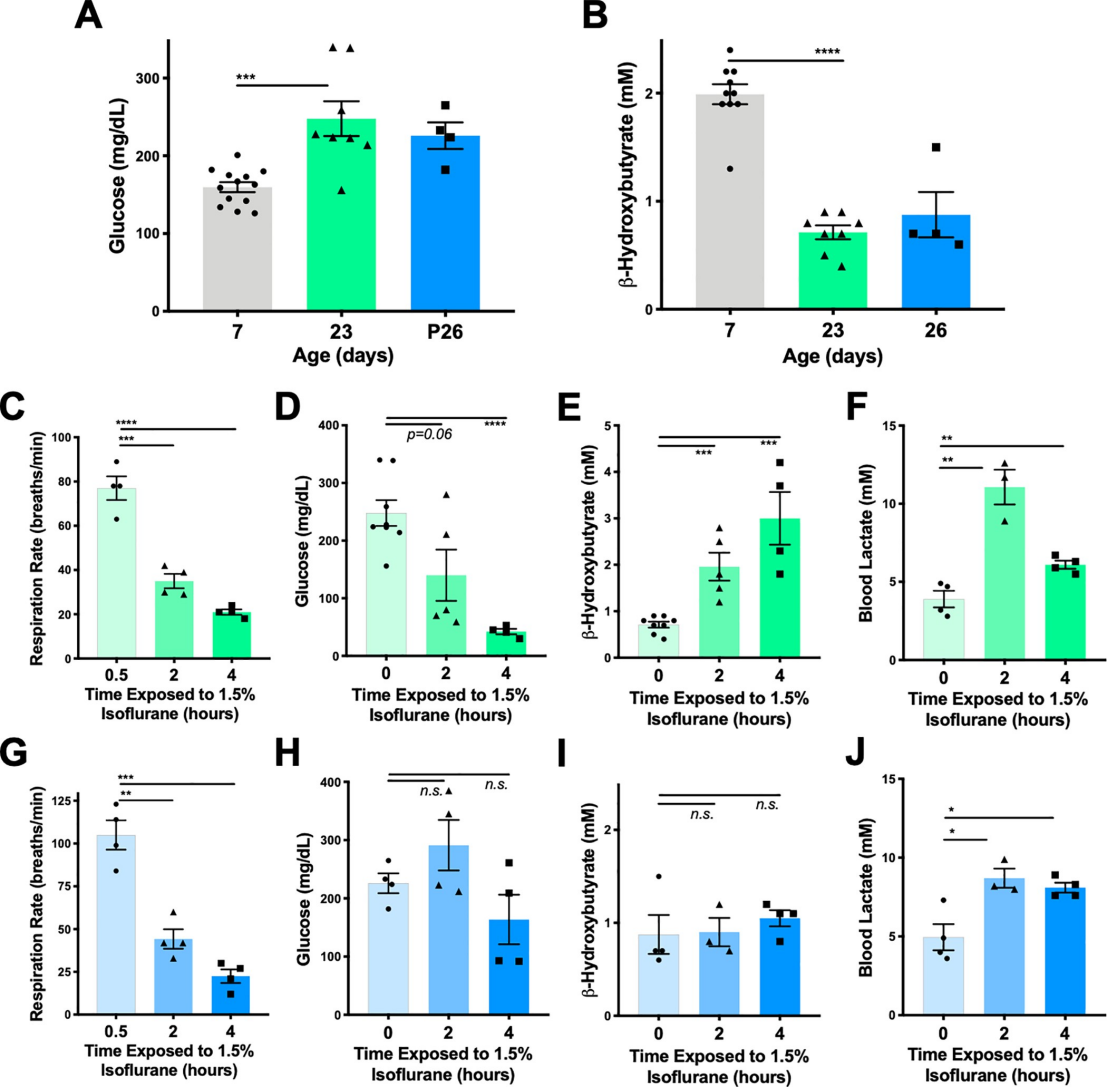
Physiological impact of anesthesia as a function of age. (**A**) Baseline blood glucose in mice at 7 (neonatal), 23 (2 days post-weaning), and 26 days of age. (**B**) Baseline blood ß-hydroxybutyrate in mice at 7 (neonatal), 23 (2 days post-weaning), and 26 days of age. (**C**) Respiratory rate of P23 mice as a function of time exposed to 1.5% isoflurane. ANOVA p-value<0.0001; ****p<0.0001, ***p<0.005 post-hoc pairwise t-test. n = 4, 4, and 4 for 0.5, 2, and 4 hours, respectively. (**D**) P23 mouse blood glucose concentration at baseline and at 2 or 4 hours of anesthesia with 1.5% isoflurane. ANOVA p-value = 0.0009; ****p<0.0001 post-hoc pairwise t-test. n = 8, 4, and 5 for 0, 2, and 4 hours, respectively. (**E**) P23 mouse blood ß-hydroxybutyrate concentration at baseline and at 2 or 4 hours of anesthesia with 1.5% isoflurane. ANOVA p-value<0.0001; ***p≤0.0004 post-hoc pairwise t-test. n = 8, 4, and 5 for 0, 2, and 4 hours of exposure, respectively. (**F**) P23 mouse blood lactate concentration at baseline and at 2 or 4 hours of anesthesia with 1.5% isoflurane. ANOVA p-value = 0.0002; **p<0.001, post-hoc pairwise t-test. n = 8, 4, and 5 for 0, 2, and 4 hours of exposure, respectively. (**G**) Respiratory rate of P26 mice as a function of time exposed to 1.5% isoflurane. ANOVA p-value<0.0001; **p = 0.001, ***p = 0.0001 post-hoc pairwise t-test. n = 4, 4, and 4 for 0.5, 2, and 4 hours, respectively. (**H**) P26 mouse blood glucose concentration at baseline and at 2 or 4 hours of anesthesia with 1.5% isoflurane. ANOVA and post-hoc t-tests non-significant (n.s.). n = 4, 4, and 4 for 0, 2, and 4 hours, respectively. (**I**) P26 mouse blood ß-hydroxybutyrate concentration at baseline and at 2 or 4 hours of anesthesia with 1.5% isoflurane. ANOVA and post-hoc t-tests non-significant (n.s.). n = 4, 4, and 4 for 0, 2, and 4 hours, respectively. (**J**) P26 mouse blood ß-hydroxybutyrate concentration at baseline and at 2 or 4 hours of anesthesia with 1.5% isoflurane. ANOVA **p = 0.006. *p<0.02 by post-hoc t-tests non-significant. n = 4, 4, and 4 for 0, 2, and 4 hours, respectively.

Studies in the mouse model have demonstrated that sensitivity to anesthetic induced neurotoxicity is specific to the neonatal period. To determine the age-range of metabolic sensitivity, *e*.*g*. the age-range where exposure to volatile anesthetics for 2 or 4 hours results in severe metabolic derangements, we exposed mice at post-natal day 23 (P23), two-days post-weaning, and post-natal day 26, 5 days post-weaning. In room air, weaned mice have higher blood glucose and lower blood ß-hydroxybutyrate than neonates (Fig 6A and 6B). When exposed to 1.5% isoflurane P23 animals show progressive respiratory depression (Fig 6C), hypoglycemia (Fig 6D), and transiently increased lactate (Fig 6F), similarly to neonates. ß-hydroxybutyrate, which starts low in P23 mice, increases significantly during a 4-hour exposure, presumably resulting from of the induction of fasting induced ketogenesis (Fig 6E). P26 mice, in contrast, show respiratory depression but non-significant changes in glucose (which trends downward) and ketones by 4 hours (Fig 6H–6J). Lactate is increased at 2 hours, but remains stable at 4. Together, these data suggest that the period of metabolic sensitivity to volatile anesthesia ends shortly after weaning, at which point mice are able to maintain metabolite levels, although some aspects of anesthesia, such as respiratory depression and lactate increase by isoflurane, are still present even at this later age.

## Discussion

Our studies demonstrate unexpected consequences of isoflurane exposure in neonatal mice. Severe derangements in respiration, blood glucose, ketones, lactate, and pH lead to a 50% mortality rate for P7 mice exposed to 1.5% isoflurane for 4 hours. Given these results, interpretation of many anesthetic neurotoxicity studies in neonatal mice must entertain confounding factors present in each study.

The majority of mouse AIN reports use 2 or more hours of anesthesia in neonatal mice, typically at P7 (neonatal mouse AIN literature summarized in **Table 1**). Several studies rely on 6 hours to induce neurotoxicity. refs Here, we show that this results in an extremely abnormal metabolic and physiological state incomparable to human clinical anesthesia. After only 2-hours P7 mouse pups show severe respiratory depression, hypoglycemia and hypoketonemia, increased blood lactate, and are severely acidotic. The extent of these metabolic defects has not been appreciated previously. By 4-hours, P7 mice are in a state of extreme metabolic and respiratory distress. Mortality reaches ~50% after a single 4-hour exposure, with animals dying during recovery from the anesthetic exposure, a clear indication that this paradigm does not model pediatric clinical anesthesia.

These data provide the most detailed assessment of the physiological impact of anesthesia in neonatal mice to date, but is supported by some published work. Loepke *et al* [18] assessed some metabolic parameters in neonatal mice exposed 1.5% isoflurane, reporting changes consistent with what we report here. They described hypoglycemia, acidosis, increased lactate, and an increase in apoptosis similar to what we found, with no cognitive defects detected. Interestingly, they used a hybrid mouse (C57Bl/6 x CD-1) for the reported ‘low’ mortality of 18% under their anesthesia protocol. These data are generally consistent with our findings, while the strain used or the carrier gas difference (30% oxygen versus 100% oxygen) may explain any differences. Although these authors do not state the overall mortality of the C57Bl/6 line in their hands they state it is higher than the 18% found with the hybrid line. Given that most mouse AIN studies utilize the C57Bl/6 background (**Table 1**) we think that our data best mimics the methods of the majority of the mouse AIN literature. It is interesting that many of the studies in Table 1 did not reported high levels of mortality, and this fact is difficult to completely reconcile with our observations. Subtle strain or colony specific effects may partly account for some variation, while differences in gas composition, or temperature control may also contribute. A comprehensive assessment of the impact of these and other potential confounding factors in neonatal rodent mortality under anesthesia may be valuable.

While we focus in this study on the neonatal mouse model of AIN, our findings are consistent with reports by Stratmann et al [19] and Wu et al [20] who found that long term exposure of neonatal rats to isoflurane or sevoflurane resulted in respiratory depression, hypercarbia, and acidosis. Importantly, these studies provided evidence that in rats the CNS cell death results from these physiological perturbations rather than the anesthesia itself. These reports also suggested that the cognitive effects of anesthetic exposure in young rats are at least partially separable from CNS cell death.

Loepke *et al* demonstrated that dextrose supplementation does not prevent CNS apoptosis in the hybrid mouse strain exposed to 1.5% isoflurane for 6 hours [18,21]. Here, we show here that in addition to hypoglycemia and acidosis, lactate is increased, respiratory rate is severely depressed, and circulating ß-HB is depleted in a much shorter exposure, only 1 hour of anesthesia. Any of these changes may contribute to CNS cell death independent of glucose, but the significant depletion of ß-HB is particularly intriguing. Neonatal mice are known to be ketotic compared to adults, and ß-HB has been shown to directly regulate histone modifier enzymes, impacting cell differentiation and survival [22–25]. Moreover, ß-HB has repeatedly been shown to prevent hypoglycemia induced neuronal damage and dysfunction in neonatal, but not adult, rodent brain slices [23,26–28]. Depletion of ß-HB has not been previously reported in the context of AIN, and loss of ß-HB may contribute to the increase in apoptosis that we see with isoflurane exposure, or to other neurodevelopmental defects associated with AIN in mice.

In these studies, we found that induction of neuronal apoptosis occurs only *after* the onset of severe systemic metabolic derangements. Moreover, despite the multiple pathologic effects of isoflurane exposure, we did not detect the extreme increase in apoptosis and CNS degeneration reported by some groups, although the degree of apoptosis induction we observed does agrees with a significant portion of the rodent AIN literature (see references in **Table 1**). In considering the variable degrees of apoptosis induction reported in the literature, we identified caspase-3 detection as a major potential source of error in studying apoptosis that are often not accounted for in published AIN reports and may contribute to lab to lab variation. We found that cleaved caspase-3 is present at extremely low levels in normal brain, and cleaved caspase-3 detection by Western blot is extremely difficult; half of the commercially available antibodies we tested detected only off-target bands (see **Methods**). Similarly, immunostaining for cleaved caspase-3 is highly sensitive to autofluorescence and off-target binding. Antibodies which recognize both the cleaved and full-length forms of caspase-3 should never be used, and the high autofluorescence of fixed brain tissue, including strong neuron body fluorescence, must accounted for. Extensive optimization led us to the conclusion that cleaved caspase-3 could not be reliably detected above background using green fluorophores so we opted for the far-red channel.

We detected no overt learning or memory deficits in mice treated with 2- or 4-hours of isoflurane, nor in animals exposed for 2-hours daily for three days. As in the case of cleaved caspase-3 induction, the published literature varies widely, many other laboratories have reported similar findings (for example [29–32]; a 2017 review by Loepke *et al* found that over a third of animal studies of AIN report either no structure or no cognitive defects [1]). Thus, the lack of overt cognitive effects in our animals indicates that our exposures paradigms were successful in modeling the published neonatal mouse AIN literature. Specifically, we found that the physiological disruptions we observed did not result in behavioral abnormalities more severe than others have reported. In considering those reports which have shown behavioral changes by Barnes maze, we did find that litter to litter variation was high in this assay, indicating that a lack of sufficient animal numbers or use of few litters could result in false positives due to litter effects. Many of the reports in Table 1 lack a detailed description of anesthesia conditions, and poorly controlled exposures may result in more severe CNS cell death and/or cognitive defects It is also possible that the mice which perished may have shown the most severe behavioral abnormalities, and that our data thus reflects a ‘survivor effect’ resulting from the loss of these animals. In this scenario, mouse strains with improved survival to 4 hours of exposure would be expected to show more overt cognitive defects, possibly accounting for some of the variance in the literature.

It is likely that isoflurane results in more subtle behavioral abnormalities than we could detect given that the Barnes maze is a relatively insensitive assay. Subtler behavioral effects are likely present following extended exposures to anesthesia but would require more sensitive or specialized assays, as others have reported (reviewed in [1]). We did not attempt to reproduce these published findings as the presence or absence of cognitive defects does not impact our primary findings.

In the clinical setting, monitoring standards for pediatric patients during anesthesia include continual monitoring of oxygenation, ventilation, circulation, and temperature both pre- and post-anesthetic exposure. Preterm neonates also usually receive intraoperative glucose to mitigate risk of hypoglycemic from low glycogen stores and immature gluconeogenesis pathways [33]. Our approach here recapitulates the ‘best controlled’ neonatal mouse AIN literature, including temperature control, 100% oxygen, humidification, and an isoflurane concentration sufficient to anesthetize but not supramaximal. Reports finding more severe apoptosis, neurodegeneration, and/or behavioral effects may partly result from less well-controlled paradigms.

Our data indicate that neonatal mouse AIN paradigms result in severe metabolic and physiological disturbances which precede CNS cell death and, accordingly, neonatal mouse AIN paradigms poorly model human clinical exposures. The work of Stratmann and of Wu [19,20] suggest this may be true for the rat model as well. However, certain aspects of AIN, such as increased CNS apoptosis and cognitive abnormalities, have been reported in larger model species where better physiological control is possible, such as Rhesus monkeys [34–36]. Accordingly, some detrimental effects of anesthesia exposure may be independent of the physiological changes and it may be possible to mechanistically separate these sequelae of anesthesia from the physiological effects in neonatal mice as well. The mouse model will remain useful in assessing the impact of anesthesia exposure if the confounding effects of physiological disruption can be avoided. Conversely, while our findings are specific to the mouse, they show that evaluation of physiological status and clinical relevance is prudent in any animal model. Given our data in mice, a careful assessment of other AIN models may be warranted.

In sum, our findings warrant a careful re-evaluation of the mouse AIN literature and the clinical relevance of mouse AIN findings.

## Supporting information

S1 Checklist Human endpoints checklist. (DOCX)

S1 File Supporting data. Contains all data presented in this manuscript. Data is organized by figure number, with each figure provided in an individual tab within the spreadsheet file.
(XLSX)
